# Insights Into the Interfacial Degradation of High-Voltage All-Solid-State Lithium Batteries

**DOI:** 10.1007/s40820-022-00936-z

**Published:** 2022-09-19

**Authors:** Jiawen Li, Yuchen Ji, Haoran Song, Shiming Chen, Shouxiang Ding, Bingkai Zhang, Luyi Yang, Yongli Song, Feng Pan

**Affiliations:** 1 School of Advanced Material, Peking University Shenzhen Graduate School, Shenzhen, 518055 People’s Republic of China; 2 Guangzhou Key Laboratory of Clean Transportation Energy Chemistry, School of Chemical Engineering and Light Industry, Guangdong University of Technology, Guangzhou, 51006 People’s Republic of China

**Keywords:** Solid-state battery, Poly(ethylene oxide), Surface modification, Interface stability, High-voltage cathode

## Abstract

Poly(ethylene oxide) (PEO)-based solid polymer electrolyte (SPE) is considered as a promising solid-state electrolyte for all-solid-state lithium batteries (ASSLBs). Nevertheless, the poor interfacial stability with high-voltage cathode materials (e.g., LiCoO_2_) restricts its application in high energy density solid-state batteries. Herein, high-voltage stable Li_3_AlF_6_ protective layer is coated on the surface of LiCoO_2_ particle to improve the performance and investigate the failure mechanism of PEO-based ASSLBs. The phase transition unveils that chemical redox reaction occurs between the highly reactive LiCoO_2_ surface and PEO-based SPE, resulting in structure collapse of LiCoO_2_, hence the poor cycle performance of PEO-based ASSLBs with LiCoO_2_ at charging voltage of 4.2 V vs Li/Li^+^. By sharp contrast, no obvious structure change can be found at the surface of Li_3_AlF_6_-coated LiCoO_2_, and the original layered phase was well retained. When the charging voltage reaches up to 4.5 V vs Li/Li^+^, the intensive electrochemical decomposition of PEO-based SPE occurs, leading to the constant increase of cell impedance and directly causing the poor performance. This work not only provides important supplement to the failure mechanism of PEO-based batteries with LiCoO_2_, but also presents a universal strategy to retain structure stability of cathode–electrolyte interface in high-voltage ASSLBs.

**Supplementary Information:**

The online version contains supplementary material available at 10.1007/s40820-022-00936-z.

## Highlights

- The cycle performance of poly(ethylene oxide) (PEO)-based all-solid-state lithium batteries with LiCoO_2_ cathode was greatly improved via coating LiCoO_2_ with high-voltage stable Li_3_AlF_6_.
- At the upper cutoff voltage of 4.2 V, the poor electrochemical performance is mainly originated from the structure collapse of LiCoO_2_ at the surface instead of the decomposition of PEO.
- When the voltage reaches 4.5 V or even higher potentials, the intensive electrochemical decomposition of PEO-based solid polymer electrolyte accelerated interfacial degradation.

**Supplementary Information:**

The online version contains supplementary material available at 10.1007/s40820-022-00936-z.

## Graphical abstract

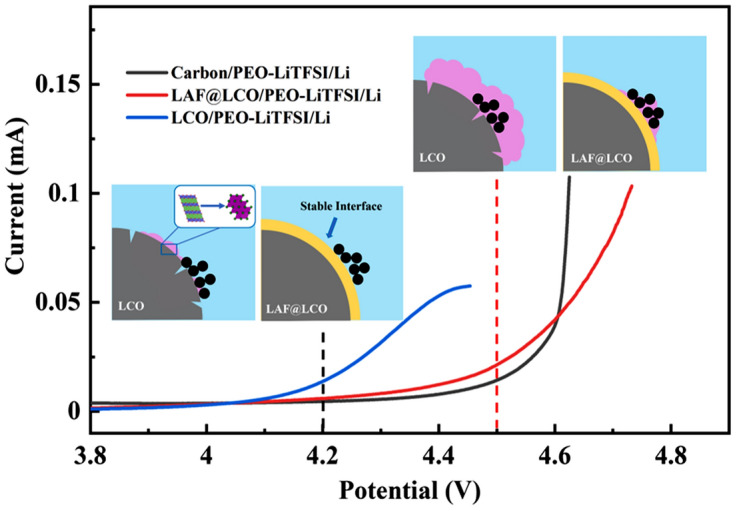

## Introduction

All-solid-state lithium batteries (ASSLBs) with solid-state electrolyte (SSE) are deemed to be the most promising alternative to conventional lithium-ion batteries owing to their improved safety and high energy density [1–9]. Among the SSE, poly(ethylene oxide) (PEO)-based solid polymer electrolyte (SPE) possesses high ionic conductivity, low cost and low interfacial resistance toward electrodes [10–14].

However, it has been widely reported that PEO-based SPE has a relatively narrower electrochemical window than other types of SSE. At charging voltage of above 3.9 V (vs. Li/Li^+^), PEO-based SPE is likely to undergo electrochemical decomposition [15–19]. When combined with high-voltage cathodes, such as LiCoO_2_, lithium nickel manganese cobalt oxide (NCM) and lithium nickel cobalt aluminum oxide (NCA), the PEO-based ASSLBs present poor electrochemical performance [20–24].

Recently, numerous efforts have been devoted to improving cycling performance of ASSLBs with PEO-based SPE and high-voltage cathodes [24–26]. Zhou et al. [27] introduced high-voltage stable poly(*N*-methyl-malonic amide) middle layer to protect PEO-based SPE from high-voltage oxidation, which stabilized the PEO-based ASSLBs operating in the voltage range of 3.0–4.25 V. Besides, Liang et al. [28] adopted lithium niobium oxide (LNO) thin film on the surface of NMC811 particles to stabilize the interface of NCM811 and electrolyte and thus suppress the oxidation of the PEO-based SPE.

Nevertheless, the failure mechanism of PEO-based SPE with high-voltage cathodes remains unclear. Yang et al. [29] tended to attribute the poor cycling performance to the structure instability of PEO-based SPE at high voltage. Meanwhile, it is supposed that the reactive terminal –OH group may be the root cause of the narrow electrochemical window of PEO-based SPE. By replacing terminal –OH group with more stable –OCH_3_, the electrochemical window could be extended to 4.3 V, which boosted the cycling stability of solid-state Li–NCM532 batteries. In another study, Chen’s group [30] concluded that electrochemical decomposition of PEO-based SPE is not the only cause for the poor performance of LiCoO_2_/PEO-LiTFSI/Li batteries. When paired with LiMn_0.7_Fe_0.3_PO_4_ cathode, the PEO-based solid-state battery shows much higher capacity retention than that with LiCoO_2_ under the same cutoff voltage (4.2 V vs Li/Li^+^). To better understand such inconsistency, a systematic study regarding to the interfacial decomposition of PEO is required.

Herein, Li_3_AlF_6_ (LAF) solid-state electrolyte is chosen as coating material on the surface of LiCoO_2_ particles to systematically analyze the failure mechanism of PEO-based solid-state batteries with LiCoO_2_ electrode. LAF was predicted to have good electrochemical stability and high ionic conductivity through theoretical calculation [31–33]. The results reveal that, at charging voltage of 4.2 V, the serious capacity decay mainly derives from structure collapse of LiCoO_2_, resulting in phase transition of the LiCoO_2_. When the charging cutoff voltage reaches up to 4.5 V or even higher, PEO-based SPE decomposed severely, leading to the constant increase of cell impedance and the degradation of battery. With the protection of LAF coating layer, structure failure of LiCoO_2_ is inhibited and the decomposition of PEO-based SPE is reduced, exhibiting improved capacity retention.

## Experiment Section

### Preparation of PEO-Based SPE

PEO (Mw = 600,000, ACROS ORGANICS), lithium bis(trifluoromethanesulfonyl)imide (LiTFSI, Aladdin, 99%) and Al_2_O_3_ were dispersed in anhydrous acetonitrile (Innochem, 99.9%) and stirred continuously for 10 h at 80 °C. The mole ratio is EO:Li = 25:1. The obtained homogeneous gel-like solution was then casted onto a Teflon substrate, and the solvent was slowly evaporated at room temperature first, and SPE was then further dried for 24 h at 80 °C in an Ar-filled glovebox to obtain PEO-based SPE.

### Preparation of LAF-coated LiCoO_2_

Firstly, LiCoO_2_ and NaF were dispersed and stirred for 4 h in deionized water. LiCl was added into the mixture solution and stirred for 6 h to obtain in situ LiF-coated LiCoO_2_. The obtained LiF-coated LiCoO_2_ and AlF_3_ were dispersed and stirred in deionized water for 8 h and then dried at 80 °C. Finally, the composite was sintered at 400 °C for 10 h to obtain LAF-coated LiCoO_2_.

### Electrochemical Measurements

LAF-coated LiCoO_2_ or bare LiCoO_2_ cathode electrodes were prepared by mixing LAF-coated LiCoO_2_ or pristine LiCoO_2_ powder, PEO, LiTFSI and SP in anhydrous acetonitrile to form a homogeneous slurry. The mass ratio of LiCoO_2_: PEO: SP was 7: 2: 1 by weight, and the amount of LiTFSI was based on the content of PEO (EO: Li = 10: 1). Then, the slurry was casted on a carbon-coated aluminum foil with doctor blade, followed by drying overnight at 110 °C in Ar-filled glove box. 2032 coin-type cells were assembled in Ar-filled glove box and constructed using bare LiCoO_2_ and LAF-coated LiCoO_2_ electrode, lithium metal anode and PEO-based SPE. The assembled cells were kept at 60 °C for 30 h before test. The cycling test was performed in the voltage range of 3.0–4.2 V (vs. Li/Li^+^) at current density of 0.2C (1C = 150 mAh g^−1^) at 60 °C. Electrochemical impedance spectroscopy (EIS) measurements were taken with an electrochemical workstation (1400 cell test system, Solartron) in the frequency range from 1 MHz to 0.1 Hz with 10 mV amplitude at 60 °C.

### Materials Characterization

The X-ray diffraction (XRD) patterns of bare LiCoO_2_ and LAF-coated LiCoO_2_ were collected by Bruker D8 Discover diffractometer with Cu Kα radiation within the 2θ range of 10–80°. The morphology and elements distribution of samples were characterized by scanning electron microscope (SEM, ZEISS SUPRA55) with an energy-dispersive spectroscopy (EDS, OXFORD, X-MaxN TSR). High-resolution transmission electron microscopy (HRTEM) images were collected using JEOL3200FS field-emission transmission electron microscopy (FETEM). All these cross-section samples were prepared by focused ion beam (FIB, FEI, Scios).

## Results and Discussion

**Fig. 1:**
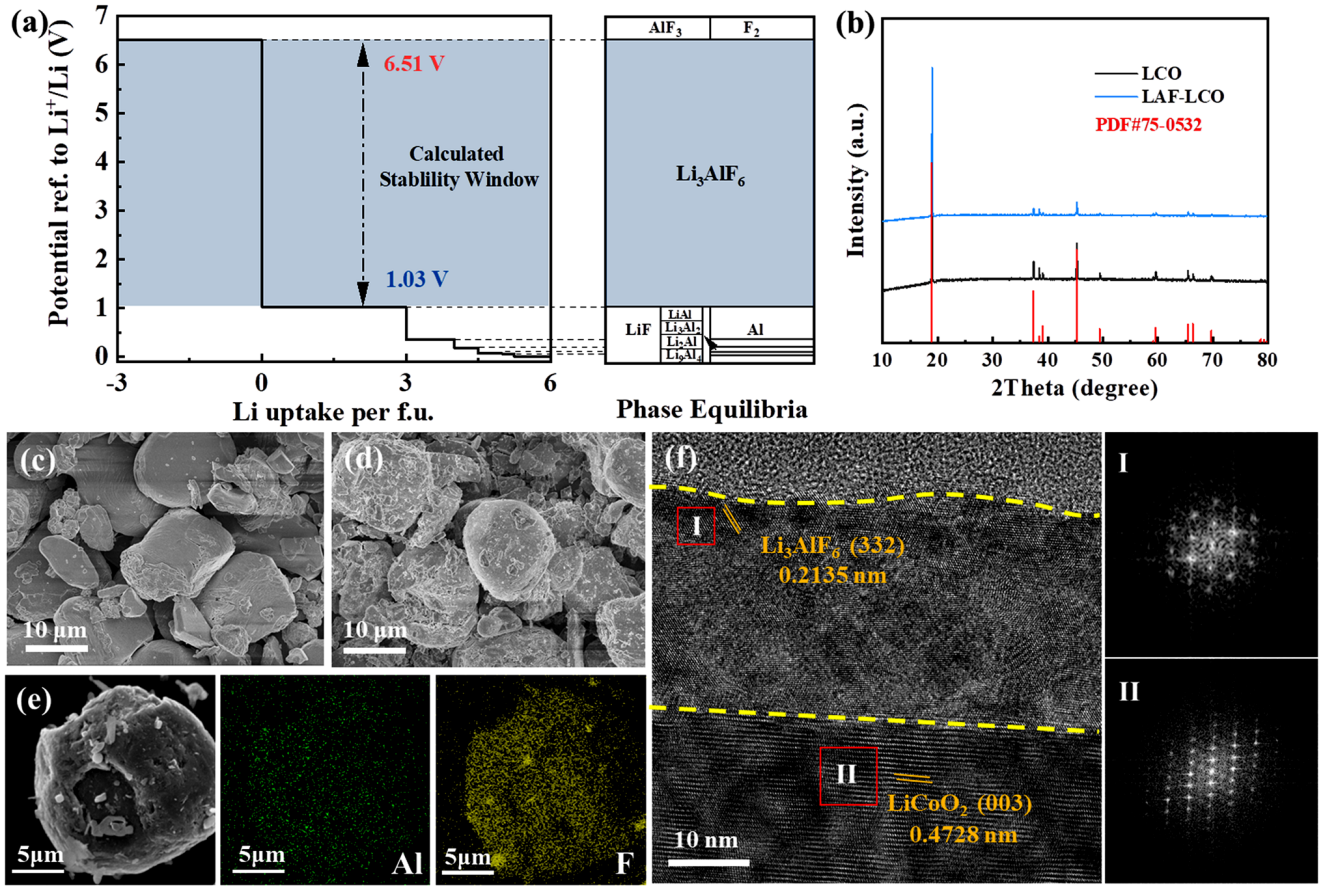
**a** Thermodynamic equilibrium voltage profile and phase equilibria of Li_3_AlF_6_. **b** XRD patterns of LCO and LAF@LCO. SEM image of **c** LCO particles and **d** LAF@LCO particles. **e** EDS elemental maps of Al and F on a LAF@LCO particle. **f** HRTEM images of LAF@LCO. (**I** and **II**) FFT patterns of marked region **I** and **II** in **f**, respectively

The thermodynamic electrochemically stable window of LAF was calculated by density functional theory (DFT) method. The equilibrium voltage profile and corresponding phase equilibria are shown in Fig. 1a. The LAF exhibits a wide electrochemical window with a cathodic limit of 1.03 V (vs Li^+^/Li) and an anodic limit of 6.51 V (vs Li^+^/Li). Meanwhile, LAF was proved to possess acceptable ionic conductivity in previous study [32]. In view of that PEO-based SPE is likely to undergo electrochemical decomposition charging voltage of above 3.9 V (vs. Li/Li^+^) and the decomposition may cause battery performance degradation [15–19]. The introduction of a modification layer with a wider electrochemical window is a common method to improve battery performance [34, 35]. Therefore, the high-voltage stable LAF thin film with acceptable ionic conductivity is suitable to modify the interface between LiCoO_2_ cathode and PEO-based SPE to demonstrate the failure mechanism of LCO/PEO solid-state battery.

The as-prepared LAF-coated LiCoO_2_ (LAF@LCO) samples show the same XRD pattern as the bare LiCoO_2_ (LCO) (Fig. 1b, JCPDS No. 75–0532), indicating the well-preserved structure of LiCoO_2_ after surface modification. The SEM images show that the relatively smooth surface of LCO becomes rough after coated with a layer of Li_3_AlF_6_ (Fig. 1c, d). In Fig. 1e, EDS mapping of LAF@LCO particle shows clear signals of Al and F element. But the bare LCO with smooth surface only exhibits signals of Co and O element (Fig. S1). HRTEM is employed to further characterize the structure of LAF layer, which was uniformly coated over the whole surface of LCO. As shown in Fig. 1f, the clear lattice fringes of bulk area in HRTEM exhibit interplanar distance of 0.4728 nm belonging to the (003) planes of the layered phase in LAF@LCO particle [36–38], which is the same as bare LCO (Fig. S2). A lattice stripe in surface area shows different interplanar distance of 0.2135 nm, close to the interplanar distance of the (332) plane of Li_3_AlF_6_ (JCPDS No. 88-0860) [39–41].

**Fig. 2:**
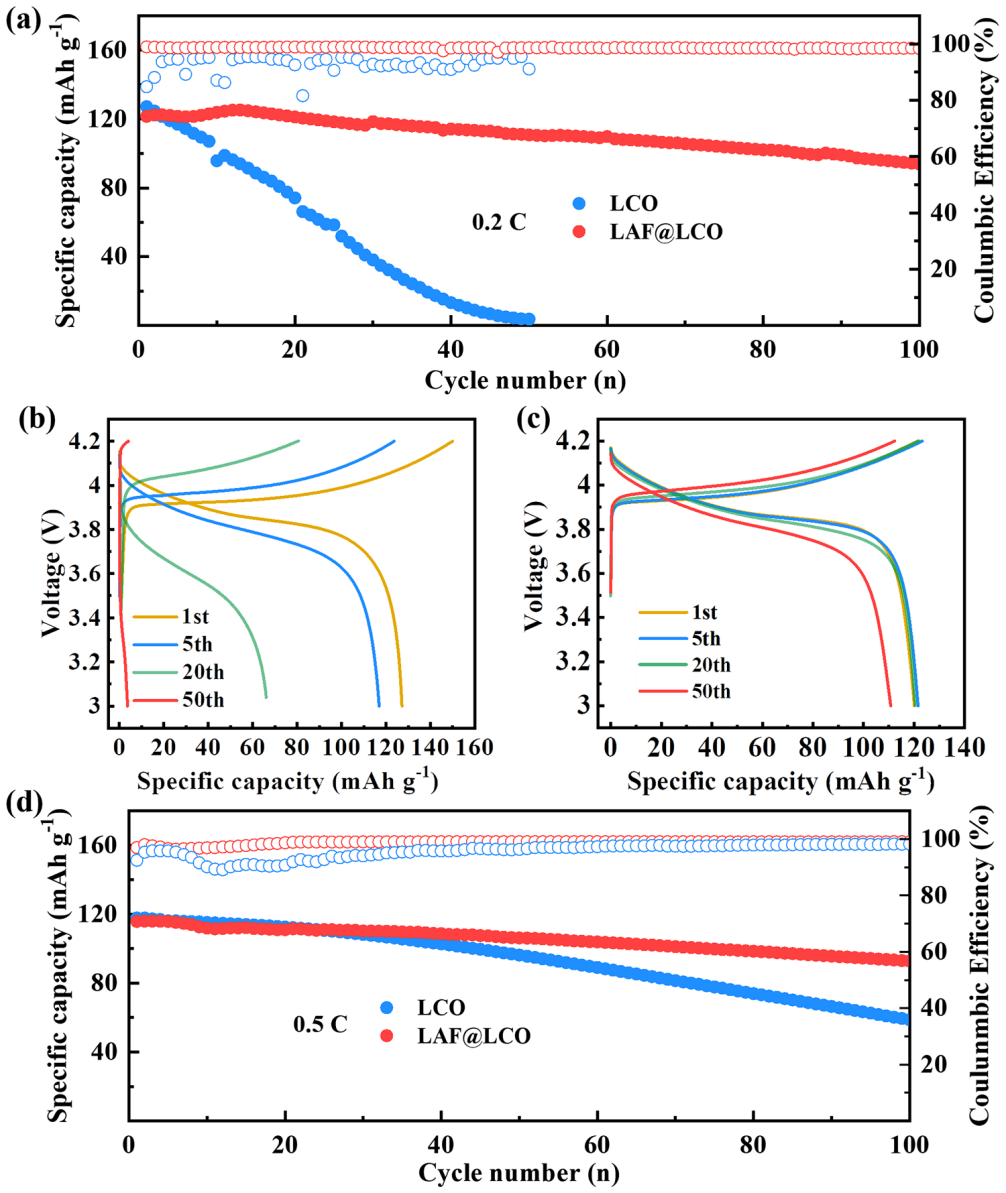
**a** The discharge capacity and Coulombic efficiency at 0.2C for 100 cycles. Charge/discharge profiles of **b** LCO/PEO-LiTFSI/Li cell, **c** LAF@LCO/PEO-LiTFSI/Li cell. **d** The discharge capacity and Coulombic efficiency at 0.5C for 100 cycles. All cells were tested at 60 °C and pre-cycled for five cycles at 0.2C

To evaluate the effectiveness of the modification strategy, the galvanostatic charge/discharge performance of LCO and LAF@LCO with PEO was tested using 2032 coin-type cells in the voltage range of 3.0–4.2 V (vs Li^+^/Li) at 0.2C at 60 °C. The cycling performance of LCO and LAF@LCO at 0.2C (Fig. 2a) shows that despite the slightly lower initial capacity, LAF@LCO delivered much-improved cycling stability as well as higher Coulombic efficiency compared with pristine LCO, which completely failed after 50 cycles. Figure 2b, c shows the charge/discharge curves of LCO and LAF@LCO at different cycles, respectively. The cycle performance of LAF@LCO is much better than that of LCO. The LAF@LCO cell shows an initial specific capacity of 122 mAh g^−1^ at 0.2C and increases to 125 mAh g^−1^, followed by slightly decrease to 98 mAh g^−1^ over 100 cycles with capacity retention of 75.1%. The initial increase of capacity could result from gradual wetting of LiCoO_2_ electrodes and PEO-based SPE [42, 43]. In contrast, the LCO cell decays rapidly from 127 to 10 mAh g^−1^ after 50 cycles. The LAF@LCO also exhibits superior cycle performance to LCO at 0.5C, with 80.2% of discharge capacity retention (Fig. 2d). The charge/discharge curves of LCO and LAF@LCO at 0.5C are presented in Fig. S3. The above results suggest that the surface modification promotes the interfacial stability between LCO and PEO-based SPE.

**Fig. 3:**
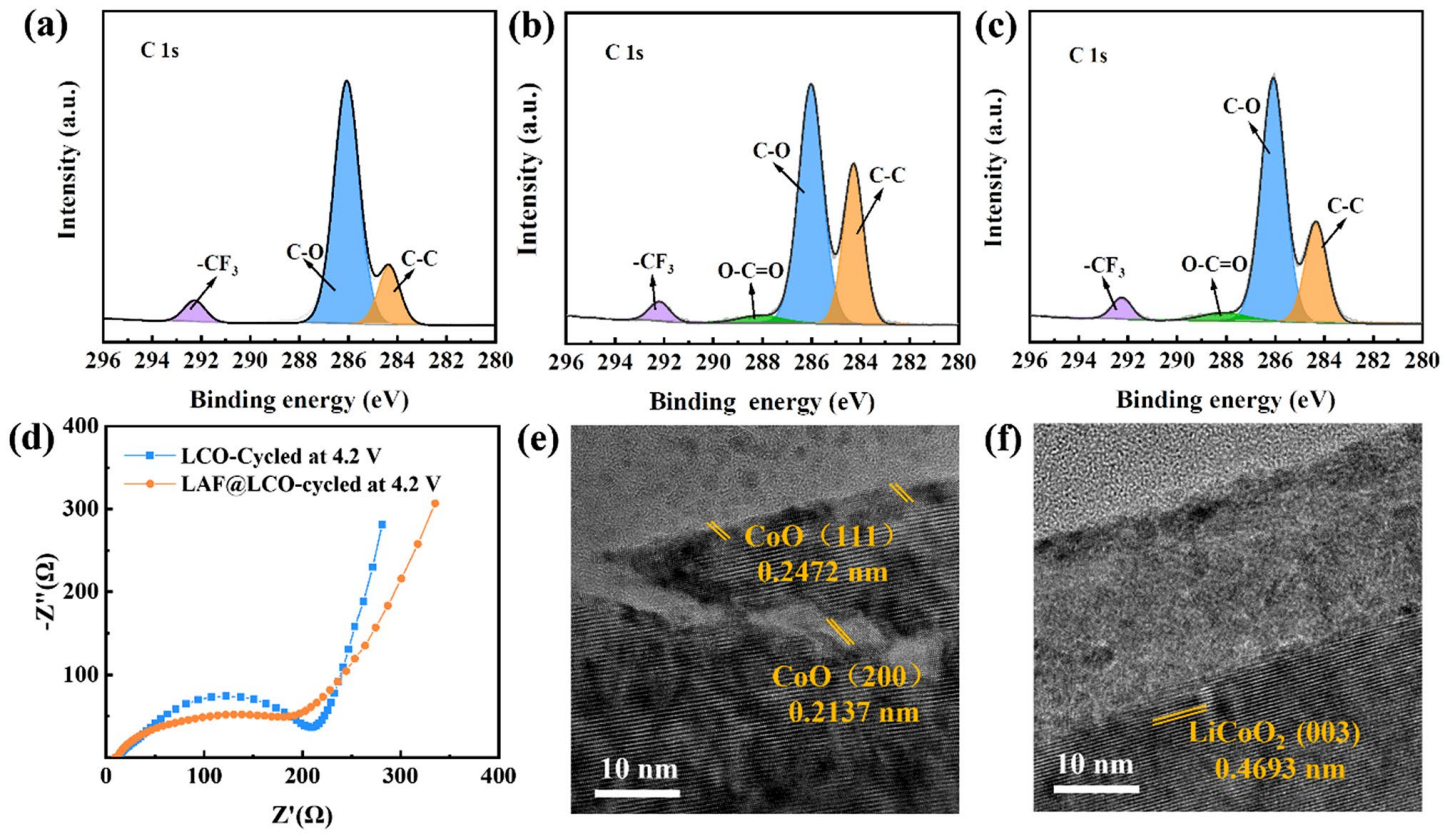
C 1*s* from **a** pristine PEO-based SPE, PEO-based SPE surface after cycling with **b** LCO electrode and **c** LAF@LCO electrode. **d** EIS spectra of LCO/PEO-LiTFSI/Li cell and LAF@LCO/PEO-LiTFSI/Li cell after cycling. HRTEM images of **e** LCO and **f** LAF@LCO particles after cycling. Cycled samples were obtained from the ASSLBs after 50 cycles in the voltage range of 3.0–4.2 V at 0.2C

To investigate the role of LAF coating layer in promoting electrochemical performance, EIS, TEM and X-ray photoelectron spectroscopy (XPS) were conducted. Figure 3a shows the C 1*s* spectra from pristine PEO-based SPE. Three peaks can be observed at 292.1, 285.7 and 284.4 eV, which can be assigned to –CF_3_, C–O, C–C, respectively [44–47]. The –CF_3_ signal in C 1*s* spectra comes from LiTFSI [48, 49]. The C–O signal in C 1*s* and O 1*s* (532.5 eV) spectra (Fig. S4a) can be attributed to the ether chain (–C–O–C–) in PEO-based SPE. The initial areal ratio of C–O to C–C peak is 1: 0.21. Figure 3b, c shows the C 1*s* spectra from PEO-based SPE surface after cycling with LCO and LAF@LCO at 3.0–4.2 V, respectively. By comparison, the areal ratio of C–O to C–C peak in C 1*s* XPS spectra decreases to 1: 0.55 after cycling with LCO electrode, which indicates the loss of ether chain. O–C=O (288.5 eV) peak can be originated from the decomposition products of PEO-based SPE, because of the redox reaction with LCO during charge/discharge process. As PEO consists of repeating –(O–CH_2_–CH_2_)– units, the emerging peak corresponding to O–C=O group (288.5 eV) group is attributed to the oxidative decomposition of PEO [50]. Previous studies have shown that the hydroxyl group (–OH) at the end of PEO molecular chain will lose electrons and form monatomic free radicals (–O), while protons are trapped by lithium salts (as shown in Eq. 1) [54]. In view of the low proportion of hydroxyl groups in the molecular chain, we have made the following assumptions regarding to the decomposition process of PEO at 4.2 V voltage: C-O bond in PEO molecular chain breaks, forming long-chain polymers with –O^·^ and –CH_2_^·^ groups, respectively (Eq. 2). The unstable –CH_2_^·^ group further converts to polymer with –CH^·^–CH_3_ (Eq. 3). –O^·^ groups will then react with –CH^·^–CH_3_ groups, forming O–C–O group (Eq. 4), which tends to be oxidized into O–C=O group under oxidative potential (Eq. 5), and protons are also believed to be trapped by lithium salts. Delithiated Li_x_CoO_2_ containing highly oxidative Co^4+^ will accelerate this reaction process by attracting electrons and protons.

12345

Herein, we use the areal ratio of O–C=O to C–O peaks in C 1s spectra as the indicator of the oxidation degree of PEO. With an upper cutoff voltage of 4.2 V, the ratios obtained from LCO and LCO@LAF after cycling are 0.08 and 0.05, respectively. indicating that the decomposition of PEO is effectively inhibited by LAF layer.

EIS was carried out to evaluate the cell impedance (Fig. 3d). However, the bulk resistance (*R*_b_) of PEO-based SPE and total impedance of cells with LCO and LAF@LCO electrode are unchanged and almost equal after 50 cycles (Fig. S5), which demonstrates that the decomposition products still possess favorable ionic conductivity during long-term cycles. It could be concluded that the decomposition of PEO-based SPE may not be the primary cause for capacity fading.

The surficial structure change of cycled LiCoO_2_ is also analyzed by HRTEM. As seen in Fig. 3e, different lattice stripes appear at the surface of LiCoO_2_ particle. The interplanar distance of 0.2472 and 0.2137 nm belongs to the (111) and (200) plane of CoO [51, 52], respectively, which indicates the structure of LiCoO_2_ is transformed into CoO phases during cycling. The structure transition of LiCoO_2_ is consistent with previous report, and CoO has been found to suppress the Li^+^ transport [30]. The phase transition unveils that chemical redox reaction occurs at the interface between the highly reactive LiCoO_2_ surface and PEO-based SPE, resulting in structure collapse of LiCoO_2_, hence the poor cycle performance of LCO at voltage of 4.2 V.

By sharp contrast, no obvious structure change can be found at the surface of LAF@LCO, and the original layered phase was well retained (Fig. 3f). It can be concluded that under this testing condition, LAF serves as a protective layer to shield LiCoO_2_ from chemical degradation and promotes the cycling stability.

Compared with PEO in contact with LCO@LAF, the direct contact between PEO and LCO accelerates the oxidation of PEO. It is believed that delithiated Li_x_CoO_2_ containing highly oxidative Co^4+^ tends to react with PEO, leading to oxygen loss in LCO lattice and degradation of PEO chains that forms decomposition products. Such chemical redox reaction will be inhibited by the electronic insulating LAF coating.

**Fig. 4:**
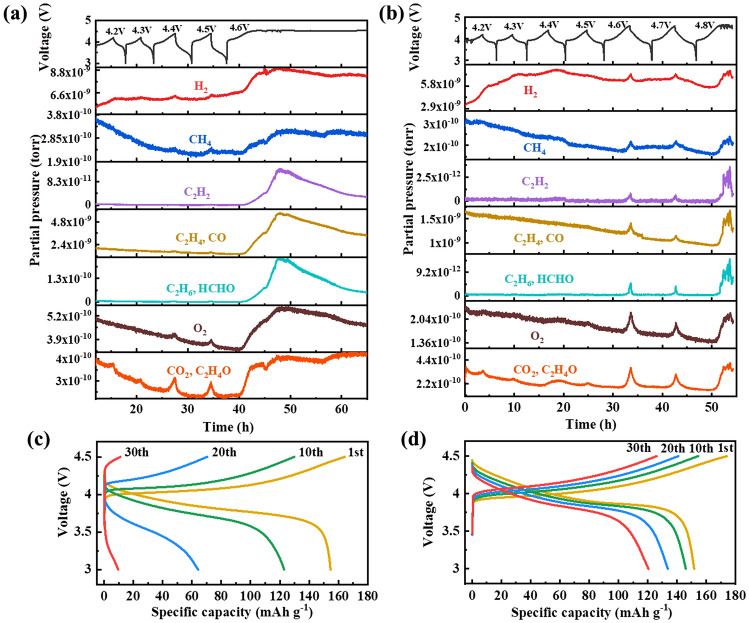
Voltage profile and corresponding in situ DEMS results of mass signals m/z 2(H_2_), 16 (CH_4_), 26 (C_2_H_2_), 28 (CO, C_2_H_4_), 30 (C_2_H_6_, HCHO), 32 (O_2_) and 44 (CO_2_, CH_3_CHO) of **a**LCO/PEO-LiTFSI/Li cell and **b** LAF@LCO/PEO-LiTFSI/Li cell cycled in the voltage ranges of 3.0–4.2, 3.0–4.3, 3.0–4.4, 3.0–4.5, 3.0–4.6, 3.0–4.7, 3.0–4.8 V for one cycle at 60 °C. A final charging to 4.8 V leads to the cell failure. The charge/discharge profiles of **c** LCO/PEO-LiTFSI/Li cell and **d** LAF@LCO/PEO-LiTFSI/Li cell. The cells are charged at a constant current density of 0.5C to 4.5 V and followed by discharging to 3.0 V at 0.2C at 60 °C

Differential electrochemical mass spectrometry (DEMS) was adopted to study the gas release behavior in PEO-based ASSLBs. LiCoO_2_/PEO-LiTFSI/Li was cycled under different upper cutoff voltages of 4.2, 4.3, 4.4, 4.5, 4.6, 4.7 and 4.8 V at 0.2C (Fig. 4a). Only trace amount of gas can be detected before 4.4 V, indicating negligible PEO decomposition. This result agrees with the conclusion drawn above that PEO decomposition might not be the reason for capacity fading of battery in the voltage range of 3.0–4.2 V. A small amount of C_2_H_4_O comes from the fracture of adjacent C-O bond in PEO as shown in Eq. 2. As the cutoff voltage gradually increases to 4.5 V, significant amounts of O_2_ are released by the cell. Before the voltage could reach 4.6 V, a long PEO decomposition plateau emerges, accompanied with severe release of various gases including H_2_, CH_4_, C_2_H_2_, C_2_H_4_, CO, C_2_H_6_, HCHO, O_2_, CO_2_ (C_2_H_4_O). In contrast, gas release started to occur at 4.6 V for LAF@LCO cells and intensive decomposition of PEO cannot be observed before 4.8 V (Fig. 4b). The improved performance may be ascribed to more stable surface of LAF@LCO particles, where PEO and LiCoO_2_ are physically separated by the LAF layer, inhibiting the chemical decomposition of PEO at the PEO-LCO interface. Moreover, in the voltage range of 3.0–4.5 V, the capacity fading of LCO cell became even faster (Fig. 4c), while the LAF@LCO cell remained relatively stable (Fig. 4d) despite a mild attenuation. The contrast of cycle performance can be observed clearly in Fig. S6. In general, the improvement of LCO/PEO performance by LAF coating in our work is satisfactory (as shown in Table S1). The DEMS data indicate that compared with 4.2 V, the decomposition reaction of PEO becomes more drastic above 4.5 V, where a large amount of gas (e.g., C_2_H_2_, H_2_, C_2_H_4_, and O_2_) are released.

**Fig. 5:**
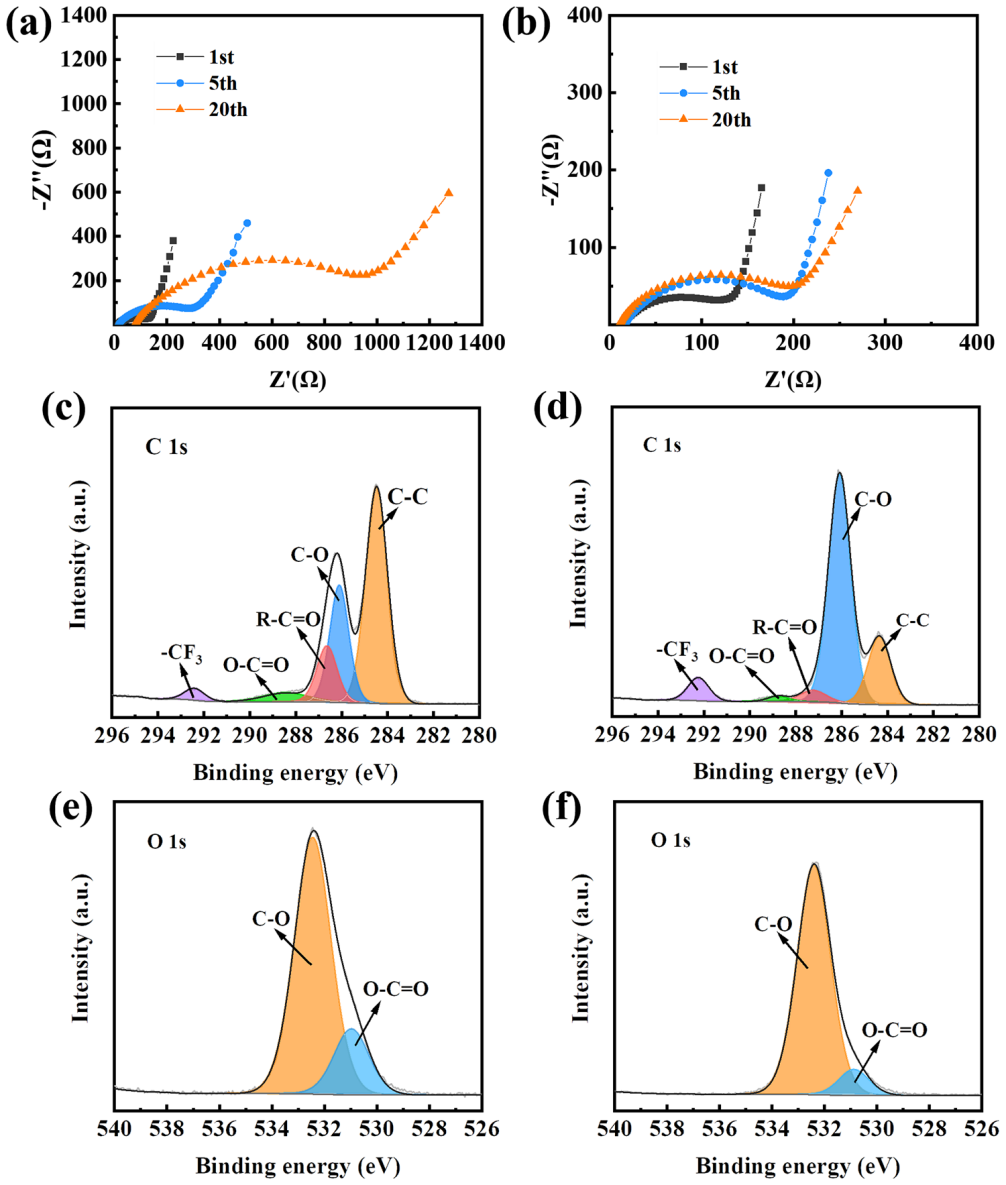
EIS spectra of **a** LCO/PEO-LiTFSI/Li cell and **b** LAF@LCO/PEO-LiTFSI/Li cell after cycling in voltage range of 3.0–4.5 V. C 1*s* from PEO-based SPE surface after cycling with **c**LCO electrode and **d** LAF@LCO electrode. O 1*s* from PEO-based SPE surface after cycling with **e** LCO electrode and **f** LAF@LCO electrode. Cycled samples were obtained from the ASSLBs after 30 cycles in voltage range of 3.0–4.5 V

The electrochemical decomposition of PEO-based SPE would lead to the increase of cell impedance. EIS is conducted to evaluate the cell impedance (Fig. 5a, b). The impedance of cell with LCO electrode increased rapidly with the increase of the cycle number and reached over 900 Ω after 20 cycles. The increase of the cell impedance reveals that continue decomposition of PEO-based SPE occurs at the interface. In contrast, the impedance of the cell with LAF@LCO electrode was retained at around 200 Ω after 20 cycles, indicating more stable interface between PEO and LAF@LCO electrode due to the presence of LAF coating layer.

XPS is carried out to characterize the electrochemical decomposition of PEO-based SPE. Figure 5c shows the C 1*s* spectra from PEO-based SPE surface after cycling with LCO. Compared with XPS data obtained from battery cycled in the voltage range of 3.0–4.2 V, a new peak corresponding to R–C=O (287.2 eV) emerged markedly after cycling with LCO electrode in the voltage range of 3.0–4.5 V, accompanied with the apparent decrease of C-O peak, which can be attributed to further oxidation of PEO segments at 4.5 V with the areal ratio of R–C = O to O–C of 0.24. This process is also accompanied with the apparent decrease of C-O peak, which can be attributed to the oxidation of PEO segments at 4.5 V. It is speculated that (shown in Eq. 6), monatomic free radicals (–O^·^) obtained previously will be further oxidized into carbonyl group [54]. In the process, delithiated Li_x_CoO_2_ containing highly oxidative Co^4+^ will also accelerate this reaction process by attracting electrons and protons. As for PEO cycled with LAF@LCO, the areal ratio of O–C = O to O–C peaks significantly decreased from 0.24 to 0.06 (Fig. 5d), suggesting a well-protected electrode–electrolyte interface at a voltage as high as 4.5 V. The same conclusion can be drawn from the O 1* s* results for both samples (Fig. 5e, f). The XPS results are in good accordance with the constant increase of impedance and the results of DEMS. Different from the capacity fading at 4.2 V, the even poorer performance at 4.5 V could be attributed to both the degradation of LCO and the decomposition of PEO.6

For LAF@LCO, the mild decomposition of PEO-based SPE could be ascribed to the deteriorated conductive carbon/SPE interface at elevated voltages. The linear sweep voltammetry results of carbon, LCO and LAF@LCO electrodes confirm the deterioration of carbon/SPE interface at high voltage. As shown in Fig. S7, the current increases abruptly at the voltage beyond 4.2 V for LCO electrode, indicating the intense decomposition of PEO-based SPE, whereas similar current increase is observed after 4.5 V for the LAF@LCO and carbon electrodes, which further demonstrates the protective effect of LAF coating layer. Since the bare carbon particles may cause the decomposition of PEO-based SPE at high voltages (≥ 4.5 V), future optimization should focus on interfacial protection at the electrode level.

## Conclusions

**Scheme 1:**
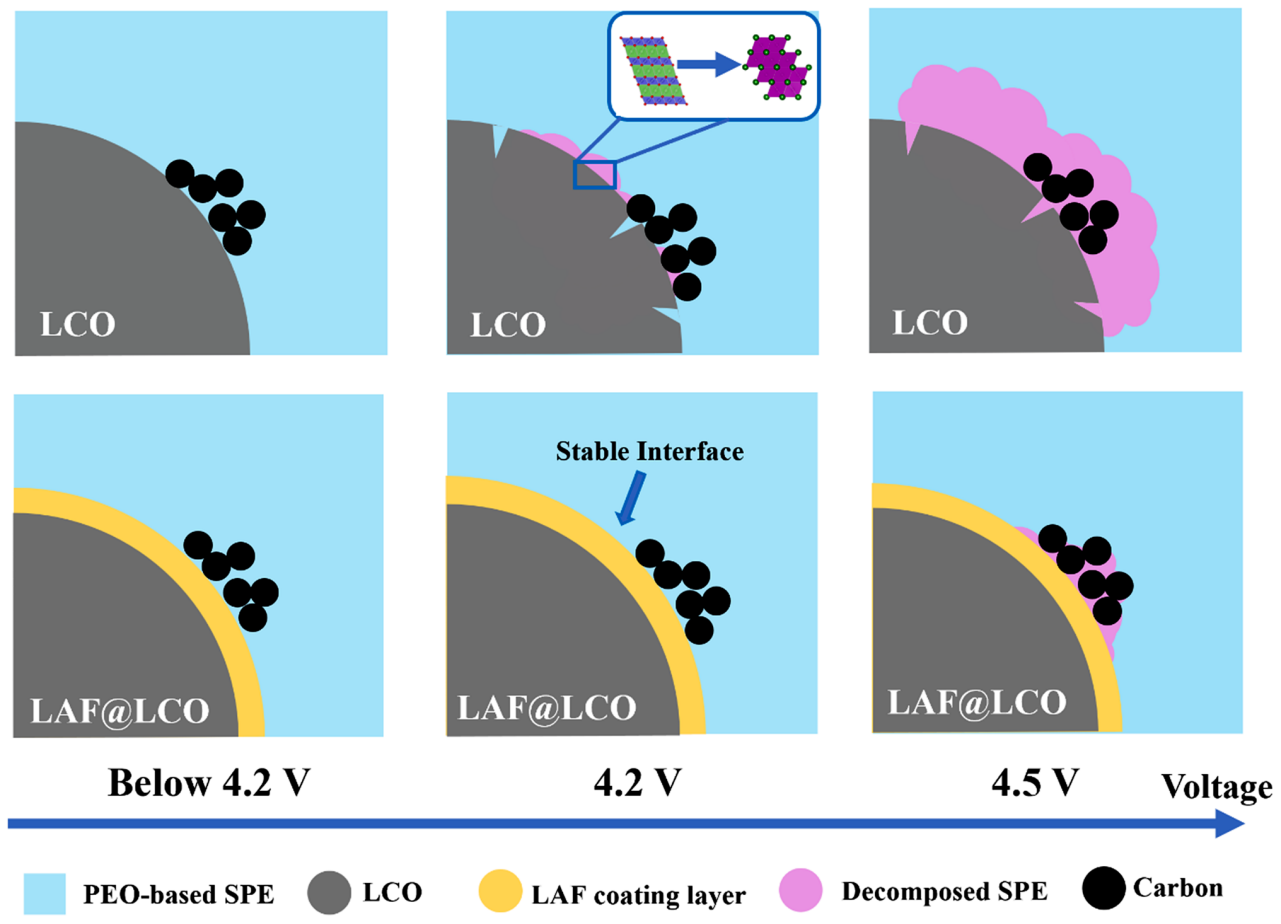
Proposed mechanism of LAF coating layer for enabling stable and high-voltage ASSLBs

In conclusion, the LAF is applied to coat on LiCoO_2_ particles for investigating the deterioration mechanism of PEO-based ASSLBs paired with high-voltage cathode. As shown in Scheme 1, at charging voltage of 4.2 V, the poor electrochemical performance is mainly originated from the structure collapse of LiCoO_2_ at the surface induced by chemical redox reaction between the highly reactive LiCoO_2_ surface and PEO, indicating that structure stability of LiCoO_2_ surface and interface is critical for high-voltage application [53]. When the voltage reaches 4.5 V or even higher potentials, both the degradation of LiCoO_2_ and the intensively decomposition of PEO could be the reasons for the capacity further fading. The results show that LAF coating layer can stabilize LiCoO_2_ by protecting them from chemical degradation during cycling and minimize the electrochemical decomposition of PEO at high voltage. This study provides an original view of the batteries failure and presents a facile strategy to extend the compatibility of PEO-based SPE with high-voltage cathode materials.

## Supplementary Information

Below is the link to the electronic supplementary material.

Supplementary file1 (PDF 676 KB)
