# Supplementary material for: Insights Into the Interfacial Degradation of High-Voltage All-Solid-State Lithium Batteries
Source: Nanomicro Lett. 2022 Sep 19;14:191. doi: 10.1007/s40820-022-00936-z (PMC9485319; doi:10.1007/s40820-022-00936-z)
Supplement: Supplementary file 1 — Supplementary file1 (PDF 676 KB) [file 40820_2022_936_MOESM1_ESM.pdf]

Supporting Information for

Insights Into the Interfacial Degradation of High-Voltage All-Solid-State Lithium Batteries

Jiawen Li[1], Yuchen Ji[1], Haoran Song[1], Shiming Chen[1], Shouxiang Ding[1], Bingkai Zhang[2], Luyi Yang[1,]*, Yongli Song[1,]*, and Feng Pan[1,]*

[1]School of Advanced Material, Peking University Shenzhen Graduate School, Shenzhen 518055, People's Republic of China

[2]Guangzhou Key Laboratory of Clean Transportation Energy Chemistry, School of Chemical Engineering and Light Industry, Guangdong University of Technology, Guangzhou, 51006, People's Republic of China

*Corresponding authors. E-mail: yangly@pkusz.edu.cn (Luyi Yang);, songyl@pkusz.edu.cn (Yongli Song); panfeng@pkusz.edu.cn (Feng Pan)

Supplementary Figures and Tables

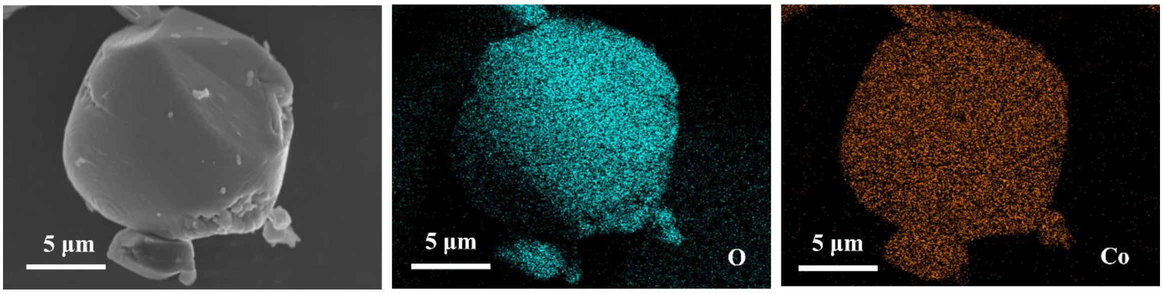

Fig. S1 EDS elemental maps of Co and O in a LCO particle

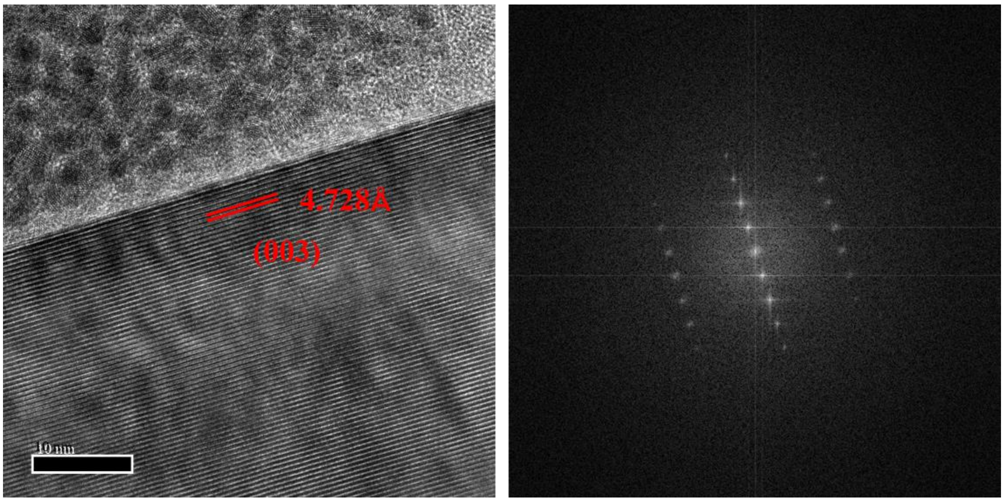

Fig. S2 TEM image and FFT pattern of LCO particle

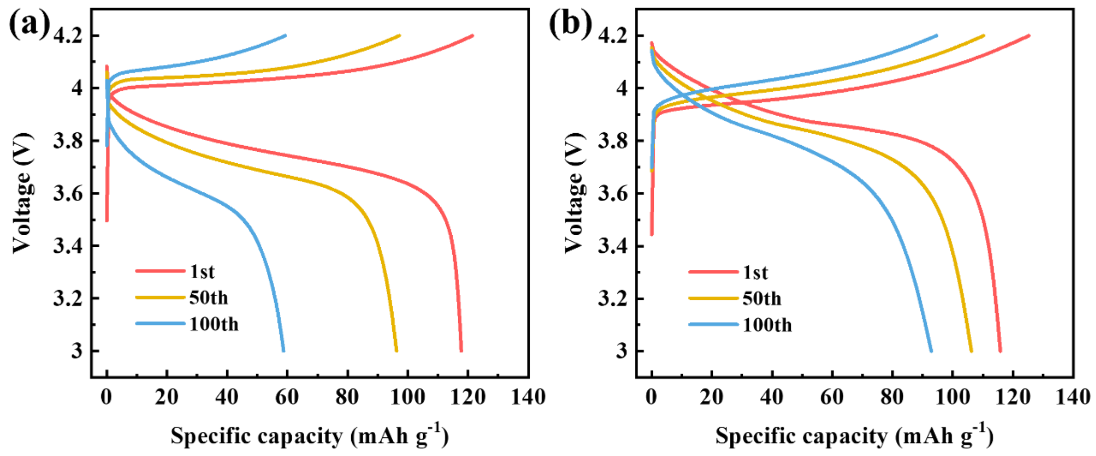

Fig. S3 Charge/discharge profiles of **a** LCO/PEO-LiTFSI/Li, **b** LAF@LCO/PEO-LiTFSI/Li cell at 0.5 C

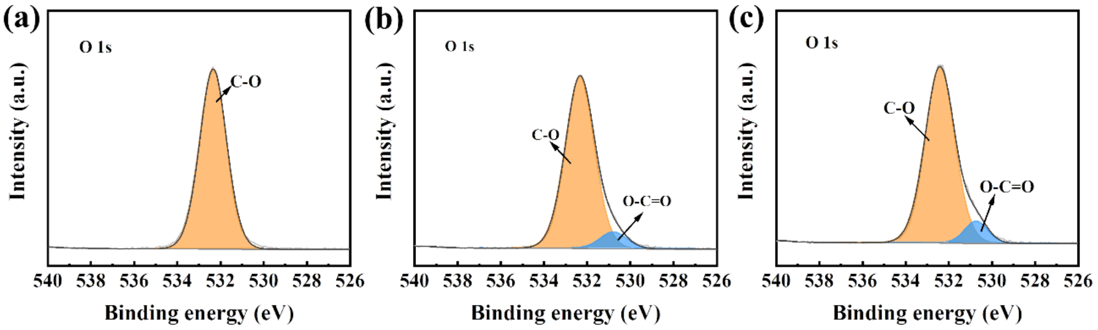

Fig. S4 O 1s from **a** pristine PEO-based SPE, PEO-based SPE surface after cycling with **b** LCO electrode and **c** LAF@LCO electrode. Cycled samples were obtained from the ASSLBs after 50 cycles in voltage range of 3.0-4.2 V

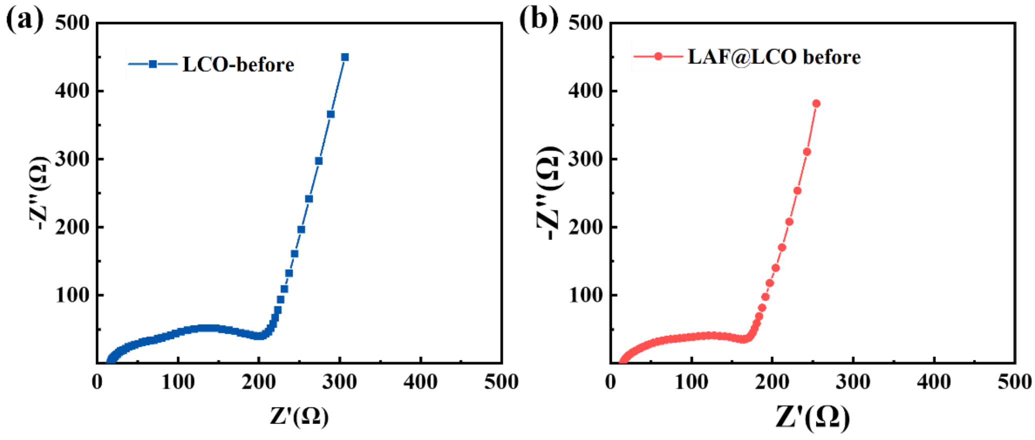

Fig. S5 EIS spectra of **a** LCO/PEO-LiTFSI/Li celland **b** LAF@LCO/PEO-LiTFSI/Li cell before cycling

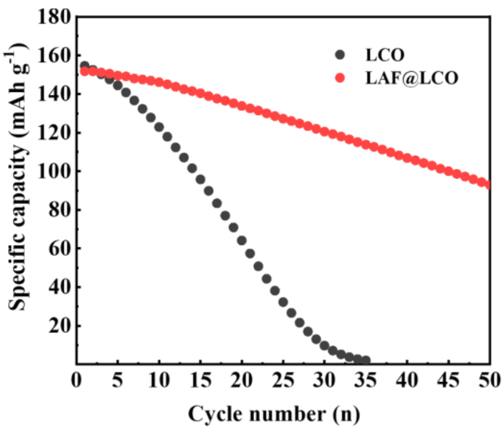

Fig. S6 The cycling performance of LCO/PEO-LiTFSI/Li and LAF@LCO/PEO-LiTFSI/Li cell, respectively. The cells were charged at a constant current of 1 C to 4.5 V, and followed by discharging to 3.0 V at 0.2 C at 60 °C

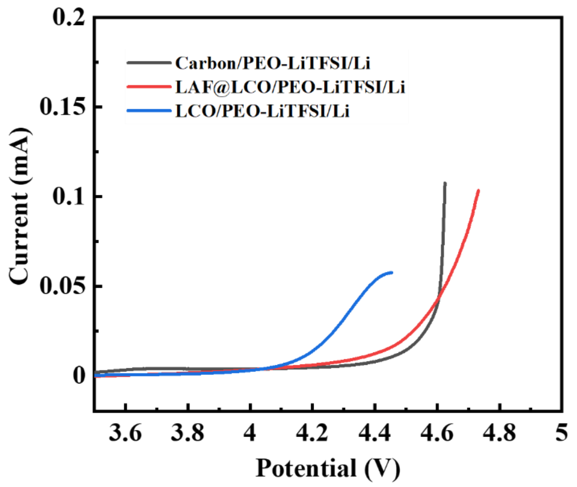

Fig. S7 Comparison of the linear sweep voltammogram of the carbon/PEO-LiTFSI/Li, LCO/PEO-LiTFSI/Li and LAF@LCO/PEO-LiTFSI/Li cell. The scan rate is 0.1 mV s^{-1}

Table S1 Performance of LCO cathode with different coating materials in PEO-based solid-state batteries

Coating material	wt% of Coating material	Cut off voltage	Capacity retention	Refs.
Li_3AlF_6	2%	4.5 V	85%/20 cycles<br>68%/50 cycles	Nano-Micro Lett. 2022, 10912
lithium tantalate		4.5 V	85%/20 cycles	J. Mater. Chem. A, 2020, 8, 2769–2776
LAGP		4.5 V	80%/7cycles	ACS Energy Lett. 2020, 5, 826–832
Li_3PO_4	5%	4.6 V	50%/9 cycles	Chem. Mater., 17, 8, 2005
PECA		4.45 V	40%/100 cycles	J. Electrochem. Soc., 2017, 164 (14), A3454-A3461
Al_2O_3	3%	4.4 V	65%/100 cycles	Chem. Mater., 2005, 17, 23
Li_3PO_4	5%	4.3 V	76%/20 cycles	Chem. Mater., 2005, 17, 8,
LATP		4.2 V	93.2%/50cycles<br>(Coulomb efficiency<85%)	J. Power Sources 2018, 388 65–70
LATP		4.2 V	88%/50cycles	Adv. Funct. Mater. 2020, 30, 1909392
Li_3InCl_6	15%	4.2 V	68.4%/200cycles	Nano Energy 2020, 76 105015
LiDFOB-PVC		4.2 V	71.5%/500cycles	Energy Storage Mater. 2020, 32 191–198
Li_3AlF_6	2%	4.2 V	92.2%/50 cycles<br>80.2%/100 cycles	Nano-Micro Lett. 2022, 10912
